# Sarcopenia as a predictor of hospitalization among older people: a systematic review and meta-analysis

**DOI:** 10.1186/s12877-018-0878-0

**Published:** 2018-08-22

**Authors:** Xiaoming Zhang, Wenwu Zhang, Conghua Wang, Wuyuan Tao, Qingli Dou, Yunzhi Yang

**Affiliations:** Department of Emergency Medicine, the Baoan Hospital affiliated with Southern Medical University, People’s Hospital of Baoan District of Shenzhen, No. 118, Longjing, Baoan District, Shenzhen, 518101 China

**Keywords:** Sarcopenia, Hospitalization, Older people, Meta-analysis

## Abstract

**Background:**

Previous cohort studies investigating the association between sarcopenia and the risk of hospitalization have been inconsistent. We performed a meta-analysis to determine if sarcopenia is a predictor of hospitalization.

**Methods:**

Prospective cohort studies that evaluated the association between sarcopenia and hospitalization in older people were identified via a systematic search of four electronic databases (PubMed, EMBASE, Science Citation Index, and the Cochrane Library). A random-effect model was applied to combine the results according to the heterogeneity of the included studies.

**Results:**

Five studies (2832 participants) were included in this meta-analysis. Pooled results demonstrated that older people with sarcopenia were at an increased risk of hospitalization (pooled hazards ratio [HR] = 1.57, 95% confidence interval [CI] = 1.26, 1.94, I^2^ = 4.5%, *P* = 0.000) compared to those without sarcopenia. Results of subgroup analyses showed that hospitalized patients with sarcopenia had a higher rate of hospitalization (HR = 2.01, 95% CI = 1.41, 2.88, *p* = 0.000) versus patients without sarcopenia. A similar result was also found in community-dwelling older people with sarcopenia versus those without sarcopenia (HR = 1.40, 95% CI = 1.05, 1.88, *p* = 0.023). In addition, the subgroup analysis for length of follow-up showed that studies with a follow-up period of 3 years or more (pooled HR = 1.52, 95% CI = 1.19, 1.94, *P* = 0.001) reported a significantly higher rate of hospitalization among individuals with sarcopenia compared to those without sarcopenia. However, this association was not found in the studies with a follow-up period of less than 3 years (pooled HR = 1.76, 95% CI = 0.90, 3.44, *P* = 0.099).

**Conclusions:**

Sarcopenia is a significant predictor of hospitalization among older individuals, and the association may not be significantly affected by the characteristics of the population or the definition of sarcopenia.

**Electronic supplementary material:**

The online version of this article (10.1186/s12877-018-0878-0) contains supplementary material, which is available to authorized users.

## Background

Sarcopenia was first described by Rosenberg [1] in 1989, who defined sarcopenia as a condition of age-related loss of muscle mass characterized by a decrease in muscle strength and physical performance. Sarcopenia is a feature of aging [2], and the prevalence of sarcopenia is reported to vary from 1 to 29% in community-dwelling older people, from 14 to 33% in long-term care, and about 10% in acute hospital care populations [3]. Currently, sarcopenia is considered a geriatric syndrome rather than a disease [4], and accumulating evidence from epidemiological studies indicates that sarcopenia may be a predictor of aging related conditions, such as functional decline and frailty [5].

The clinical relevance of sarcopenia has been extensively studied in the past few decades, and these studies have demonstrated that older people with sarcopenia have an increased risk of multiple adverse health outcomes, which include falls [6] and fractures [7], decreased mobility [8], depression [9], poor quality of life [10], hospitalization [11] and mortality [12]. In particular, hospitalization is an important outcome for older people, because even a short-term stay in a hospital for an older people is associated with a decline in functional ability [13].

Recently, Bianchi [14] and colleagues reported that sarcopenia was significantly associated with hospitalization (hazard ratio [HR] 1.57; 95% CI 1.03–2.41) among community-dwelling older adults. Furthermore, Yang [15] and Gariballa [16] reported that sarcopenia was significantly associated with re-hospitalization, with HR: 2.49; 95% CI: 1.25–4.95 and HR: 2.52; 95% CI: 1.34–4.75, respectively in hospitalized patients. However, two previous longitudinal studies did not show a significant association between sarcopenia and hospitalization in older individuals. Cawthon [17] and colleagues found that none of the summary definitions or the definition components (slowness, weakness, or low lean mass) was associated with risk of hospitalization (Rate ratio [OR] = 1.27, 95% confidence interval [CI] = 0.85, 1.90) among 1516 males with sarcopenia compared to males without sarcopenia. In addition, Henwood [18] and colleagues reported no significant association between a baseline diagnosis of sarcopenia and hospitalization among 273 male and female nursing homes residents.

In view of these inconsistencies, a meta-analysis of the associated risk is warranted. Recently, Beaudart [19] performed a meta-analysis and systematic review to assess the association between sarcopenia and various health outcomes. However, only one study was available to estimate the association between sarcopenia and hospitalization, and the author analyzed and described the outcomes without a meta-analysis. Therefore, in this meta-analysis, we aimed to include all available prospective cohort studies to summarize the current understanding of the association between sarcopenia and risk of hospitalization in older adults.

## Methods

This systematic review was conducted and reported according to the Preferred Reporting Items for Systematic Reviews and Meta-Analyses (PRISMA) guidelines [20].

### Data sources and search strategy

A systematic literature review was conducted via searches of PubMed, EMBASE, Science Citation Index, and the Cochrane CENTRAL Library through November 2017. The search strategy was tailored to fulfill the criteria of each database. The search terms included [Hospitalization (Medical Subject Heading (MeSH)] OR (Hospitalization*) AND [(Sarcopenia (Medical Subject Heading (MeSH)] OR (Sarcopenia*). We searched the potential gray studies through Google Scholar. Furthermore, a manual search was carried out on the references of included studies to identify potentially relevant studies. The search string is included in detail in Additional file 1.

### Study selection

The studies identified by our search strategy were reviewed by two independent investigators (Zhang XM, Zhang WW) who evaluated each title and abstract of the records. In cases of disagreement about inclusion or exclusion of studies, this issue was resolved by discussion with a third investigator until consensus was reached.

### Inclusion and exclusion criteria

The inclusion criteria were as follows: prospective cohort studies. Since these studies could provide a longitudinal association between sarcopenia and the risk of hospitalization with less bias, we only included prospective cohort studies, studies aiming to investigate whether sarcopenia is a predictor of hospitalization, studies clearly reporting diagnostic criteria for sarcopenia, and studies clearly reporting outcomes of hospitalization or readmission. Exclusion criteria were as follows: insufficient data, conference abstracts or review articles, no clear definition of sarcopenia, studies using only components or subdomains of sarcopenia criteria (e.g., muscle mass, Muscle Strength, Physical Performance, SARC-F Questionnaire), and studies published in other languages than English.

### Data extraction

Two authors (Zhang XM, Zhang WW) independently extracted data from the included studies into a standardized Microsoft Excel spreadsheet. Any disagreement was resolved by consensus with a third investigator. The following data were extracted from the studies: year of publication, country, demographics of participants (e.g., sample size, and male proportion), follow-up period, definitions of sarcopenia, definitions of hospitalization outcome, and Newcastle–Ottawa scale scores. All data were cross-checked by the investigators. Disagreements were resolved via discussion until consensus was reached. Moreover, our meta-analysis was based on secondary data; therefore, the ethical approval or patient consent was not necessary.

### Assessment of risk bias

Assessment of risk of bias was performed by two independent investigators (Zhang XM, Wang CH) according to the Newcastle Ottawa Scale (NOS) [21], which evaluates the quality of cohort studies via the following aspects: representativeness of the exposed cohort, comparability of the groups, blinding of investigators who measured outcomes, the time and completeness of follow-up, contamination bias, and other potential sources of bias. A higher score indicated better quality. A detailed description is shown in Table 3 as a supplement.

### Statistical analysis

The STATA version 11.0 (Stata Corp, College Station, TX, USA) was used for all analyses. Hazard ratios (HRs), odds ratios (ORs), and 95% confidence intervals (CIs) for the incidence of hospitalization in older adults with sarcopenia were compared to those without sarcopenia. If the incidence of sarcopenia was less than 10%, we regarded ORs as RRs; otherwise, we used generic inverse variance to calculate the pooled HR. Subgroup analyses according to length of follow-up and type of participants (hospitalized, community-dwelling or nursing home residents) were implemented to analyze the association between sarcopenia and hospitalization. Statistical heterogeneity among the included studies was examined using Cochran’s Q statistic using chi-square and I^2^ Statistics, and I^2^ value of 25%, 50% and 75% represented the cut-off values for the low, moderate, and high heterogeneity, respectively. From the perspective of research and design, there was heterogeneity because of the different evaluation methods of sarcopenia, different types of participants, and length of follow-up. Therefore, we used the random-effects model to synthesize all data, regardless of heterogeneity between the pooled studies, in statistical order to obtain more conservative results. Subgroup analyses according to different populations and length of follow-up were performed to analyze the association of sarcopenia and hospitalization.

## Results

### Selection processes

**Fig. 1:**
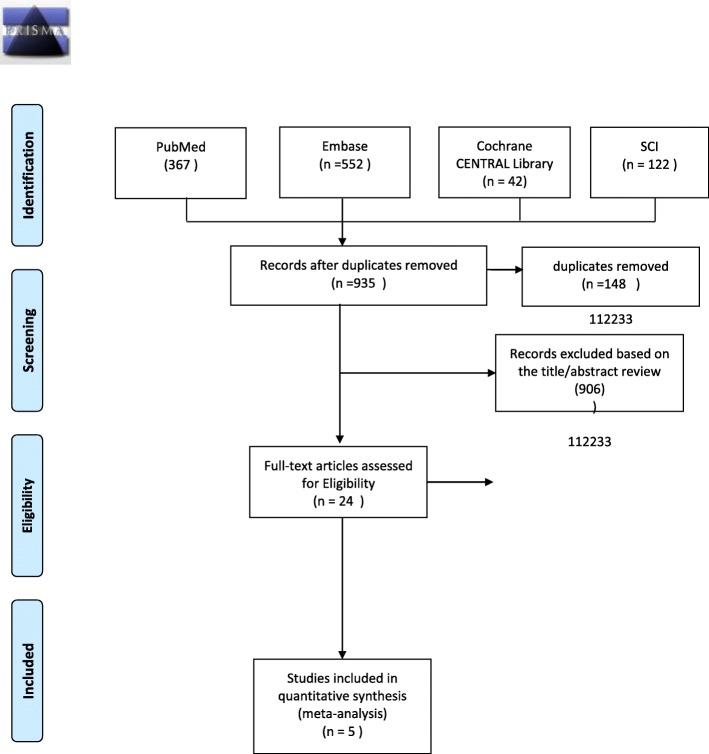
The flow diagram of studies selection

A total of 1083 articles were initially identified via our literature search. After eliminating duplicate articles, 935 articles were screened for potential eligibility. After screening the titles and summaries, non-related articles were removed and the remaining 24 articles were included for further investigation. Of these articles, six were removed because they were not cohort studies (e.g., review articles, cross-sectional study), three were removed because of irrelevant outcomes, eight were removed because they had no clear definition of sarcopenia, and two were same cohort studies. Figure 1 shows the flow diagram of our literature search and selection process.

### Included studies

Table 1 presents the characteristics of five cohort studies [14–18] included in our meta-analysis. One studies was from America [17], one was from China [15], and the remaining three were from the UK [16], Australia [18], and Italy [14], respectively. Four studies [14–16, 18] included both male and female participants, while one study [17] included only male participants. The population included in our study included hospitalized patients [15, 16], community-dwelling older people [14, 17], and nursing home residents [18]. Sample size varied from 58 to 1516 and the mean age of the study participants was > 70 years. Moreover, all of the studies selected hospitalization [14, 17, 18] or re-hospitalization [15, 16] as the clinical outcomes. In four of the studies [14, 16–18], sarcopenia was diagnosed by the European Working Group for Sarcopenia in Older Persons (EWGSOP); in one study [15], sarcopenia was diagnosed by the Asian Working Group for Sarcopenia (AWGS). The EWGSOP [22] defined sarcopenia as ALM adjusted for height squared as ≤7.26 kg/m^2^ in men and ≤ 5.54 kg/m^2^ in women combined with low hand-grip strength (< 30 kg) and low gait speed (< 0.8 m/s), and/or low grip strength < 30 kg for men and < 20 kg for women. According to AWGS [23], participants were classified as having sarcopenia if the participants had a lower muscle mass with a low handgrip strength of < 26 kg in men and < 18 kg in women, and a lower gait speed < 0.8 m/s. Follow-up periods ranged from 0.5 to 10 years. The largest study [17] involved 1516 participants, while the minimum cohort [18] involved 58 participants. The prevalence of sarcopenia ranged from 7.5 to 40.2%. The results of each study were displayed in our studies. Table 2 shows the different tools and cut-off of muscle mass, muscle strength, and physical performance.

**Table 1:** Characteristics of included studies

First author	Year	Country	Male	Sample size	Age (years)	^b^Definitions of sarcopenia	Follow-up durations	Result of effect size	Outcome	Quality^a^
P.M Cawthon	2017	USA	100%	1516	70–80	EWGSOP	3 years	OR:1.27 (0.85, 1.90)	hospitalization	7
S.Gariballa	2013	UK	74.30%	432	70–80	EWGSOP	0.5 years	HR:2.52(1.34,4.74)	re-hospitalization	6
L.Bianchi	2016	Italy	46.80%	538	77.1	EWGSOP	10 years	HR:1.57(1.03,2.41)	hospitalization	7
T.Henwood	2017	Australia	29.30%	58	80–90	EWGSOP	1.5 years	RR:1.27(0.73,2.22)	hospitalization	6
M.Yang	2017	China	78.10%	288	81.1 ± 6.6	AWGS	3 years	HR:1.81(1.17,2.80)	re-hospitalization	8

^a^Quality of the studies were assessed with Newcastle-Ottawa Scale (NOS);

^b^EWGSOP (European Working Group for Sarcopenia in Older Persons) defines sarcopenia in men as ALM adjusted for height squared < 7.25 kg/m2 combined with low hand-grip strength (< 30 kg) and/or low gait speed (< 0.8 m/s)

Asian Working Group for Sarcopenia (AWGS): participants had a lower muscle mass with a low handgrip strength of < 26 kg in men and < 18 kg in women, and/or a lower gait speed ≦ 0.8 m/s

OR, odds ratio; RR, risk ratio; HR, hazard ratio

**Table 2:** Study and sarcopenia criteria

study	Sarcopenia criteria	Item,tool, Cutoff points						Prevalence (%)	Ref
		Muscle mass		Muscle strength		Physical performance			
		Tool	Cutoff points	Tool	Cutoff points	Tool	Cutoff point		
P.M Cawthon	EWFSOP	ALM/height2	Men ALM/height^2^: ≤ 7.23 kg/m^2^)	Grip strength:	GS < 30 k	Gait speed	≤ 0.8 m/s	7.5	[17]
S.Gariballa	EWFSOP	MAMC	Men: MAMC <21.1 cm Women: MAMC <19.2 cm	Grip strength:	Men:GS<30 kg Women: GS<20 kg	None	None	10	[16]
L.Bianchi	EWFSOP	BIA	Men:SMI < 8.87 kg/m^2^ Women:SMI < 6.42 kg/m^2^	Grip strength:	Men: BMI ≤ 24 kg/m^2^ GS ≤29 kg, BMI 24.1–28 kg/m^2^ GS ≤30 kg, BMI > 28 kg/m^2^ GS ≤ 32 kg; women: BMI ≤ 23 kg/m2 GS ≤17 kg, BMI 23.1– 26 kg/m2 GS ≤17.3 kg, BMI 26.1–29 kg/m2 GS ≤18 kg, BMI > 29 kg/m^2^ GS ≤21 kg)	Gait speed:4 m	≤ 0.8 m/s	10.2	[14]
T.Henwood	EWFSOP	BIA	Men:SMI < 8.87 kg/m^2^ Women:SMI < 6.42 kg/m^2^	Grip strength	Men:GS<30 kg Women: GS<20 kg	Gait speed: 4 m	≤ 0.8 m/s	40.2	[18]
M.Yang	AWGS	SMI	men: SMI < 6.70 kg/m^2^, women: SMI < 4.75 kg/m^2^	Grip strength	Men:GS<26 kg Women: GS<18 kg	Gait speed: 4 m	≤ 0.8 m/s	17	[15]

*ALM* appendicular lean mass, *ASM* appendicular muscle mass, *AWGS* Asian Working Group for Sarcopenia, *BIA* bioimpedance analysis, *BMI* body mass index, *DXA* dual-energy X-ray absorptiometry, *EWGSOP* the European Working Group on Sarcopenia in Older People, *GS* grip strength, *MAMC* mid-arm muscle circumference, *SMI* skeletal muscle mass index

### Quality assessment

Table 3 shows the detailed description of the methodological quality evaluation using NOS. Scores ranged from six to eight and three studies reported scores > 7.

**Table 3:** Result of the Newcastle-Ottawa Scale quality assessment

Newcastle-Ottawa scale		P.M Cawthon 2017 [17]	S.Gariballa 2013 [16]	L.Bianch 2016 [14]	T.Henwood 2017 [18]	M.Yang 2017 [15]
Selection(4)	Representativeness of the exposed cohort	1	1	1	0	1
	Selection of the non-exposed cohort	1	1	1	1	1
	Ascertainment of exposure	1	1	1	1	1
	Demonstration that outcome of interest was not present at start of study	1	1	1	1	1
Comparability(2)	Comparability of cohorts on the basis of the design or analysis	1	0	0	1	2
Outcome(3)	Assessment of outcome	1	1	1	1	1
	Was follow-up long enough for outcome to occur	1	0	1	0	0
	Adequacy of follow up of cohorts	1	1	1	1	1
Quality(9)	Total	8	6	7	6	8

### Sarcopenia as a predictor of hospitalization

#### Meta-analysis of studies

**Fig. 2:**
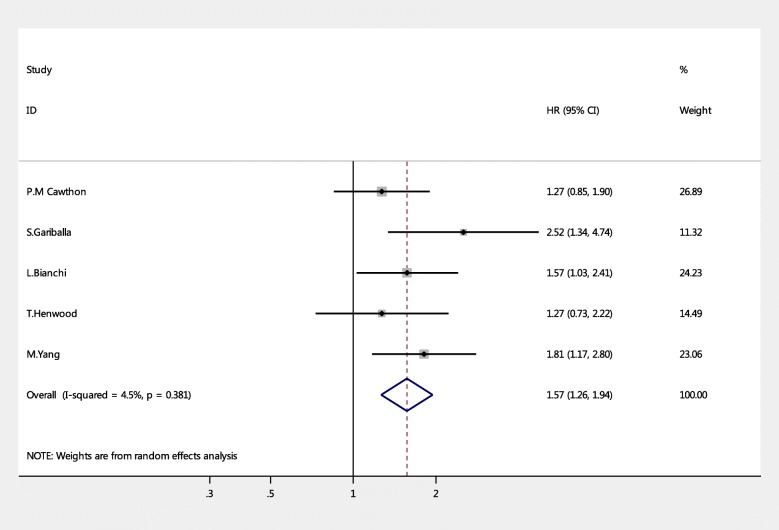
Forest plots for the meta-analysis of the association between sarcopenia and hospitalization

Five studies [14–18] evaluated the association between sarcopenia and hospitalization. Because of the different evaluation methods of sarcopenia, different type of participants, and length of follow-up, and considering the presence of the heterogeneity of the study itself, we used the random-effects model to synthesize all data, regardless of heterogeneity between the pooled studies, in statistical order to obtain more conservative results. The data are shown in Fig. 2. The pooled results showed that older people with sarcopenia had significantly higher risk of hospitalization (pooled HR = 1.57, 95% CI = 1.26, 1.94, *P* = 0.000) versus participants without sarcopenia. No significant heterogeneity was observed in these studies (Q-value = 4.19, degree of freedom = 4, I^2^ = 4.5%, *P* = 0.381).

#### Subgroup analyses

The HR of hospitalization risk for sarcopenia in five studies [14–18] was further assessed by subgroup analyses. According to the type of the participants, the association between sarcopenia and hospitalization was consistent in studies with hospitalized patients (pooled HR = 2.01, 95% CI = 1.41, 2.88, *P* = 0.004) [15, 16] and studies with community-dwelling individuals (pooled HR = 1.62, 95% CI = 1.26, 2.09, *P* = 0.000) [14, 17], but not individuals living in nursing homes (pooled HR = 1.27, 95% CI = 0.73, 2.21, *P* = 0.400) [18] with a between subgroup *p*-value of 0.23. In addition, the subgroup analysis for length of follow-up showed that studies with a follow-up period of 3 years or more [14, 15, 17] (pooled HR = 1.52, 95% CI = 1.19, 1.94, *P* = 0.001) indicated a significantly higher rate of hospitalization in participants with sarcopenia compared to those without sarcopenia. However, this association was not found in the studies with a follow-up period of less than 3 years [16, 18] (pooled HR = 1.76, 95% CI = 0.90, 3.44, *P* = 0.099). All the subgroup analyses are displayed in Table 4.

**Table 4:** Subgroup analyses of the meta-analysis

Subgroup	Effect model	Number of studies	Mean difference (95% CI)	P for Heterogeneity	I-squared Value	*P* Value for effect	*P* Value Between Groups
Type of participent	Random effect						
hospitalized patients		2	2.01[1.41, 2.88]	0.40	0%	0.0001	
community-dwelling individuals		2	1.40[1.05, 1.88]	0.48	0%	0.02	
nursing homes residents		1	1.27 [0.73,2.21]			0.40	*P* = 0.23
Follow-up	Random effect						
> = 3 years		3	1.52[1.19, 1.94]	0.496	0%	0.0007	
< 3 years		2	1.76[0.90, 3.44]	0.111	60.7%	0.10	*P* = 0.69
Outcome	Random effect						
hospitalization		3	1.37[1.06, 1.78]	0.74	0%	0.02	
re-hospitalization		2	2.01[1.41, 2.88]	0.40	0%	0.0001	*P* = 0.09
NOS score	Random effect						
≥7		3	1.52[1.19, 1.94]	0.496	0%	0.0007	
<7		2	1.76[0.90, 3.44]	0.111	60.7%	0.10	*P* = 0.69

#### Publication bias assessment

We could not conduct publication bias assessment due to the small number of the studies included.

## Discussion

This meta-analysis of five available cohort studies showed that older adults with sarcopenia have an increased risk of hospitalization. Moreover, results of subgroup analyses showed that the association between sarcopenia and increased risk of hospitalization is consistent in older people from hospital- or community-based data and in studies with different diagnostic criteria of sarcopenia. Our results highlight that sarcopenia is an important determinant of hospitalization outcome in older people, and preventative strategies against sarcopenia may improve the outcome of hospitalization for older people [24].

Beaudart [19] assessed the association between sarcopenia and various health outcomes in 2017. However, only one study was available that estimated the association between sarcopenia and hospitalization, and the author analyzed and described the outcomes without a meta-analysis. Compared to this previous systematic review, our meta-analysis included five cohort studies and focused on all hospitalizations. To the best of our knowledge, our study is the most comprehensive review summarizing cohort studies that included all definitions of sarcopenia and hospitalization.

In our systematic review, the prevalence rates of sarcopenia varied from 7.5 to 40.2%. Diversity in our analysis was mainly due to the characteristics of the older adults and the different diagnostic methods of sarcopenia. Subgroup analyses of types of participants indicate that the association between sarcopenia and hospitalization was consistent in studies with hospitalized patients (pooled HR = 2.01, 95% CI = 1.41, 2.88, *P* = 0.004) and studies with community-dwelling individuals (pooled HR = 1.62, 95% CI = 1.26, 2.09, *P* = 0.000). Although the HR of hospitalization was more than the HR of community-dwelling individuals, there was no significant difference between the groups. Generally, community-dwelling individuals were relatively healthy and had well-preserved functional ability compared to hospitalized patients. This can be attributed to the fact that hospitalized patients [15, 16] often presenting with multiple comorbidities and activated inflammatory conditions, which can result in higher levels of inflammatory markers, such as C-reactive proteins (CRP) and cytokines. In addition, according to a recent meta-analysis, sarcopenia is associated with higher serum inflammatory parameters, particularly increased CRP levels [25]. Therefore, more prospective cohort studies are needed to compare hospitalized patients with community-dwelling older people. There was only one study [18] that addressed nursing home residents. That study included 102 older adults at baseline from 11 nursing homes; 58 of these older adults were followed-up for 18 months. The author concluded that there were no statistical significant association between sarcopenia and hospitalization in nursing home residents (pooled HR = 1.27, 95% CI = 0.73, 2.21, *P* = 0.400).

Subgroup analysis for length of follow-up indicated that a follow-up period of 3 years or more resulted in significantly higher rates of hospitalization in participants with sarcopenia compared to those without sarcopenia. However, this association was not found in the studies with a follow-up period of less than 3 years. The difference in results may be attributed to aging or sarcopenia-aggravated hospitalization [26], which may increase the rate of hospitalization. This suggests that if sarcopenia is detected early, it can potentially be managed nutritionally or with physical activity to reduce the hospitalization probability.

The underlying mechanisms driving the association of sarcopenia and risk of hospitalization were unable to draw conclusions. Some results of previous studies provide potential explanations. First, sarcopenia has been associated with poor endurance, physical disability, slow gait speed, and decreased mobility [6]. Besides disability, muscle strength is also a strong predictor of hospitalization [27, 28], and older people with sarcopenia tend to have low muscle mass or poor physical performance [29], which increases their risk of hospitalization. Secondly, multiple studies [30–33] found that sarcopenia is a significant predictor of future falls among older adults. When older people fall, they can suffer hip, spine, hand, and/or pelvic fractures, which could increase the probability of hospital admission. According to the US Centers for Disease Control and Prevention, there were 2.5 million nonfatal falls among older adults presenting to the emergency department, 734,000 hospitalizations, and a direct medical cost of $30 billion in 2013. [34] Thirdly, sarcopenia is multifactorial, in that it can be associated with a range of ages [35], multiple chronic health conditions, unhealthy lifestyle [36], deregulated hormonal factors [37], inflammation [38] and so on [39]. All of these factors are considered risk factors for hospitalization and can exacerbate sarcopenia. In addition, sarcopenia has been considered a predictor of future fracture in older adults [40–42], which raises the risk of subsequent hospitalization. As a geriatric syndrome rather than a disease, the underlying mechanisms for the pathogenesis of sarcopenia may be complex. Intensive studies are needed to clarify the potential mechanisms underlying the association between sarcopenia and hospitalization. Of the utmost importance, prevention of the multiple factors underlying sarcopenia, particularly nutrition and physical activity, may be very important. There is a growing body of literature that recognizes specific physical activity programs as the most important approach to slowing the decline of muscle mass and muscle strength associated with aging and to treat sarcopenia [43]. Adequate nutrition [44], specifically protein and micronutrients (vitamin D), has to be regarded as an essential requisite for any successful therapeutic approach for prevention and treatment of sarcopenia. By taking preventative measures to decrease the rate of sarcopenia, we can further decrease the medical expenses and resources related to hospitalization.

There are some strengths and limitations in our systematic review and meta-analysis. One of the strengths is that we performed an extensive search of electronic data-bases and evaluated the quality of each study. The primary limitation of our analysis is the limited number of longitudinal studies and the small sample sizes of the studies, which may have affected the quality of our systematic review and the results of subgroup analyses. Secondly, we only included studies published in English and, consequently, may have overlooked data from important studies published in other languages, which may have resulted in potential bias. Third, although we combined adjusted results, we could not exclude the residual confounding factors that may affect the association between sarcopenia and hospitalization risk.

## Conclusion

This meta-analysis suggests that sarcopenia is associated with increased risk of hospitalization of community-dwelling and hospitalized older adults. These results indicate that we should more closely monitor older adults in hospitals and community-dwelling locations. Comprehensive diagnosis and treatment of sarcopenia should be conducted in hospitalized older people to reduce the progression of sarcopenia and improve prognosis. Early detection of sarcopenia is beneficial in community-dwelling older people, and appropriate nutrition and exercise interventions are necessary to reduce the harmful consequences of sarcopenia and ultimately reduce the risk of hospitalization of older adults.

## Additional file

Additional file 1: Search strategy of PubMed research report. (DOCX 13 kb)

## Abbreviation

AWGS: Asian Working Group for Sarcopenia
EWGSOP: European Working Group on Sarcopenia in Older People definition
NOS: Newcastle-Ottawa scale
