# Supplementary material for: Sarcopenia as a predictor of hospitalization among older people: a systematic review and meta-analysis
Source: BMC Geriatr. 2018 Aug 22;18:188. doi: 10.1186/s12877-018-0878-0 (PMC6103964; doi:10.1186/s12877-018-0878-0)
Supplement: Supplementary file 1 — Search strategy of PubMed research report. (DOCX 13 kb) [file 12877_2018_878_MOESM1_ESM.docx]

# Additional file 1: Search strategy of PubMed research report

MEDLINE (via PubMed)

#1: Search sarcopeni*

#2: Search "Sarcopenia"[Mesh]

#3: Search ("Sarcopenia"[Mesh]) OR sarcopeni*

#4: Search "Hospitalization "[Mesh]

#5: Search Hospitalization*

#6: Search readmissions

#7: Search hospital readmissions

#8: Search re-hospitalized

#9: ((((hospitalization) OR Hospitalization*) OR readmissions) OR hospital readmissions) OR re-hospitalized

#10: (((sarcopeni*) OR "Sarcopenia"[Mesh])) AND (((((hospitalization) OR Hospitalization*) OR readmissions) OR hospital readmissions) OR re-hospitalized)
